# Thyroid incidentalomas on 18FDG-PET/CT: a metabolico-pathological correlation

**DOI:** 10.1186/s40463-017-0200-8

**Published:** 2017-03-21

**Authors:** N. Hagenimana, J. Dallaire, É. Vallée, M. Belzile

**Affiliations:** 1 Division of Otorhinolaryngology and Head & Neck Surgery, Department of Surgery, Université de Sherbrooke, Sherbrooke, Québec Canada; 2 Department of Nuclear Medicine, Université de Sherbrooke, Sherbrooke, Québec Canada

**Keywords:** Thyroid incidentalomas, ^18^F-fluorodeoxyglugose Positron Emission Tomography with computed tomography, Malignancy risk, Algorithm, SUV

## Abstract

**Background:**

Thyroid incidentaloma is defined as an unsuspected thyroid lesion found on imaging study or while performing a surgery non-related to the thyroid gland. Most recent scientific literature tends to demonstrate a detection rate of 0.1–4.3% for incidental findings of thyroid focal uptake identified by ^18^F-fluorodeoxyglugose Positron Emission Tomography with computed tomography (^18^FDG-PET/CT) initially prescribed for nonthyroid disease. From 10.3 to 80.0% of patients who underwent further evaluation are diagnosed with malignant lesions.

Our first objective is to determine the risk of malignancy confined in thyroid incidentalomas(IT) detected on ^18^FDG-PET/CT in patients treated in a tertiary care center (Centre Hospitalier Universitaire de Sherbrooke). Second, we want to identify a cut-off value for SUVmax in order to distinguish benign from malignant IT. Third, we look for predictive criterion that can be outlined to help in their management.

**Methods:**

We retrospectively reviewed 40 914 charts of patients who had a ^18^FDG-PET/CT done in a tertiary center from 2004 to 2014. For each patient where a thyroid incidentaloma has been identified, Maximum Standardized Uptake Value (SUVmax), ultrasound report, cytology and histopathological results as well as oncologic outcomes were compiled and analyzed.

**Results:**

In this study, the incidence for thyroid incidentaloma detected with ^18^FDG-PET/CT is 0.74%. The rate of malignancy present in IT is 8.2% based on histopathological results. Of the patients who underwent surgery, thyroid malignancy was identified in 54.3% of them. Cytoponction showed a strong correlation with final histopathological results (*p = 0.009)*.

**Conclusion:**

Thyroid incidentalomas detected with ^18^FDG-PET/CT are relatively infrequent, but the potential risk of malignancy remains elevated. Fine needle aspiration biopsy is the investigation of choice to rule out a malignant incidentaloma when there is no other element in the clinical portrait to preclude such additional work up.

## Background

Thyroid incidentaloma (TI) is defined as a thyroid gland lesion fortuitously discovered during radiology exams, like computed tomography or ultrasound. This type of lesion can also be identified during a neck surgery unrelated to the thyroid. ^18^F-fluorodeoxyglugose Positron Emission Tomography with computed tomography (^18^FDG-PET/CT) is a nuclear medicine imaging technique based on glucose hypermetabolism from malignant cells. It is indicated mostly for detection and follow up in patients with malignancies. In this regard, ^18^FDG-PET/CT for detection of malignancies amongst thyroid focal uptake has a sensitivity of 100%, a specificity of 69%, a positive predictive value (PPV) of 62% and a negative predictive value (NPV) of 100% [1]. Other studies report values ranging from 60 to 80% for sensitivity and from 66.1 to 91.0% for specificity [2]. Iagaru et al.*,* regarding a different patient population with confirmed thyroid carcinoma, stated that ^18^FDG-PET/CT has a high sensitivity (88.6%) and specificity (89.3%) when used for follow up [3].

The popularity of the ^18^FDG-PET/CT leads to an increasing number of thyroid incidentalomas, or any other type of unexpected site of hypermetabolism interpreted as suspicious for malignancy. As thyroid glucose uptake can be nonspecific, the prevalence of malignancies amongst thyroid incidentalomas is still uncertain. While a recent meta-analysis identified a rate of malignancy of 19.8% [4], in other studies, the prevalence of TI detected by ^18^FDG-PET/CT ranged from 0.1 to 4.3% and the risk of malignancy stands between 10.3 and 80.0% [5–12].

In this retrospective study, we benefit from Sherbrooke’s significant experience with ^18^FDG-PET/CT to review the data related to thyroid incidentalomas and to estimate the risk of malignancy for such lesions. The secondary objective was to determine a threshold value for the Maximum Standardized Uptake Value (SUVmax) where a TI could be considered as malignant. We also aimed to generate a clinical management algorithm for this day-to-day situation.

## Methods

### Patients

Between August 1^st^ 2004 and August 1^st^ 2014, a total of 40 914 ^18^FDG-PET/CT were done in the Centre Hospitalier Universitaire de Sherbrooke (CHUS). Of those, 1369 patients were extracted after we questioned CIRESS data bank with the keywords in french «thyroid», «thyroid nodule» and «thyroid incidentalomas» to be written in the nuclear medicine final report. We individually reviewed the 1369 charts and, from those, 304 patients with thyroid incidentalomas were identified. The remaining 1065 files were excluded on the basis of the following exclusions criteria:

(i) Known thyroid nodule or thyroid disease documented in the final report or in the patient’s chart
(ii) ^18^FDG-PET/CT specifically done for thyroid disease
(iii) Absence of documentation available in our institution in pre- or post-^18^FDG-PET/CT patient’s chart.

For each patient with a thyroid incidentaloma, the presence of complementary investigations, including neck ultrasound and fine-needle aspiration cytology results were noted. For neck ultrasound, we stratified the results as low risk, suspect or malignant. In the ATA guidelines 2015, ultrasonographic criteria suspicious for malignancy are: presence of microcalcifications, nodule hypoechogenicity compared with the surrounding thyroid or strap muscles, irregular margins (defined as either infiltrative, microlobulated or spiculated) and a shape taller than wide measured on a transverse view [13]. Based on final report from radiologist, ultrasound was low risk when no criteria or only hypoechogenicity was encountered. A suspect ultrasound had two or three criteria mentioned and malignant ultrasound had four suspicious features.

FNA results were reported according to Bethesda system for reporting of thyroid cytopathology: Bethesda 1 (Non-diagnostic/Unsatisfactory) lied in the non-diagnostic category. Bethesda 2 and 3 (Benign or AUS/FLUS) were put in the low risk category. Bethesda 4 (Follicular neoplasm) was stratified as intermediary and Bethesda 5 and 6 (Suspicious for malignancy and Malignant) were put in the high risk category [14]. Finally, when available, the histopathological results were also compiled as benign or malignant.

### ^18^FDG-PET/CT

All the ^18^FDG-PET/CT exams were done according to the standard nuclear medicine department protocol in our institution. If the size(cm) of the TI or its SUVmax was missing, the exam was re-read by the nuclear medicine specialist attached to this study.

### Statistical analysis

The Mann–Whitney *U* Test or a logistic regression analysis were used with continuous variables to determine if they were predictive values for malignancy in thyroid incidentalomas. The exact test of Fisher or a Chi^2^ (*χ* ^2^) were used for dichotomous or categorical variables. Data were analyzed by IBM SPSS Statistics 20. A *p* value less than 0.05 determined the threshold of a statistically significant difference. Air under the curve (AUC) was used from a ROC curve to identify a SUVmax cut-off value.

## Results

**Fig. 1:**
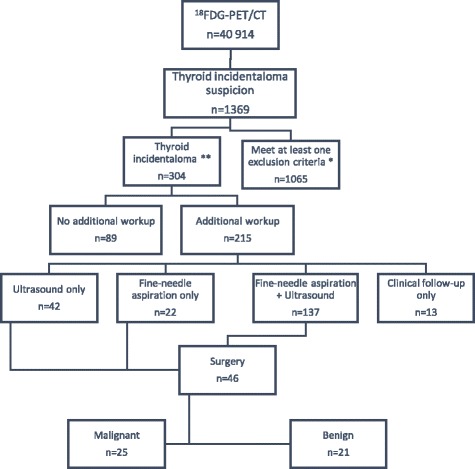
Flow diagram for patients with Thyroid Incidentalomas (TI) at the CHUS, between 2004 and 2014

**Fig. 2:**
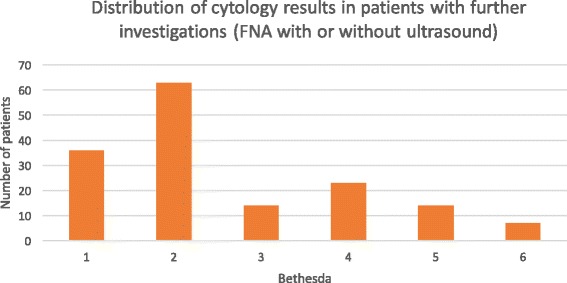
Cytology results in patients who underwent FNA

Of the 40 914 ^18^FDG-PET/CT done in Sherbrooke between 2004 and 2014, 304 (0.74%) thyroid incidentalomas were identified. Amongst these 304 TI, further evaluation, including a medical follow-up, an ultrasound and/or a fine-needle aspiration (FNA), was performed in 215 of them (Fig. 1). Hundred and fifty-nine patients underwent an FNA and the results based on the Bethesda system are illustrated in the Fig. 2. One patient from the “ultrasound only” group and one in the “clinical follow up” group went for surgery, 5 from the “FNA only” group and 39 from the “ultrasound and FNA” group. Histopathologic confirmation from surgery was obtained in 46 patients. Of those, 21 were low risk based on the FNA, 5 were intermediate and 18 were high risk. Two surgical patients did not have any FNA done preoperatively. Twenty-five patients out of 46 (54.3%) were confirmed with a malignant thyroid lesion: 18 papillary carcinomas, 4 follicular carcinomas, 1 anaplastic carcinoma of the thyroid, 1 metastasis of a neuroendocrine tumor and 1 non-hodgkinian lymphoma B-cell subtype.

Clinical characteristics of patients with a comparison of ^18^FDG-PET/CT findings and histopathological results of operated patients are shown in Table 1. After statistical analysis, sex (male) and fine-needle aspiration results were the potent predictors of thyroid malignancy (*p* value = 0.008 and 0.002, respectively). Neither SUVmax nor thyroid incidentaloma size measured in cm on TEP/CT were predictors of malignancy (*p* value = 0.499 and 0.873, respectively). Neck ultrasound failed to be within statistical significance.

**Table 1:** Clinical characteristics of surgical patients with thyroid incidentalomas (*n* = 46)

Characteristics	Malignant	Benign	*p*
	(*n* = 25)	(*n* = 21)	
Age (years)	61.88 ± 12.07	61.86 ± 10.27	0.996
Sex (M/F)	14/11	4/18	0.008
SUVmax	4.90 [2.95–8.65]	4.40 [3.55–7.73]	0.499
Size^a^ (cm)	1.50 [1.10–2.25]	1.45 [1.13–1.68]	0.873
Ultrasound^b^			0.264
Low risk *n* (%)	5 (38.5)	8 (61.5)	
Suspect *n* (%)	13 (54.2)	11 (45.8)	
Malignant *n* (%)	3 (100.0)	0 (0.0)	
Cytoponction^c^			0.009
Low risk *n* (%)	3 (30.0)	7 (70.0)	
Intermediate *n* (%)	6 (46.2)	7 (53.8)	
High risk *n* (%)	14 (82.4)	3 (17.6)	
Non diagnostic *n* (%)	2 (33.3)	4 (66.6)	

^a^From the PET/CT

^b^Based on radiology report of suspicious features for malignancy according to ATA guidelines 2015 (Low risk = no suspicion or hypoechogenic nodule with no other features, suspect = 2 or 3 suspicious features, malignant = 4 suspicious features)

^c^According to the Bethesda system for reporting thyroid cytopathology

Data are shown as Mean ± standard deviation, Median [IQR] unless otherwise indicated

**Fig. 3:**
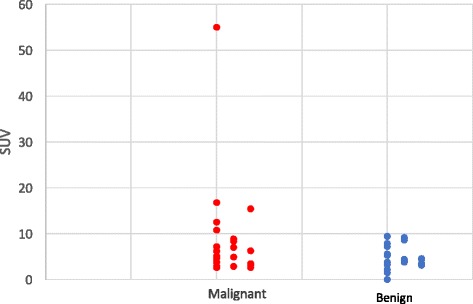
SUV values of thyroid incidentalomas identified with 18FDG-PET/CT compared to the histopathologic results (*n* = 46)

Figure 3 represents the distribution of SUVmax whether the lesion was benign or malignant on final histopathological report. Highest SUVmax in malignant nodule was 55.0 while it reached 9.4 for benign incidentalomas. Every thyroid incidentalomas (*n* = 3) with a SUVmax value ≥10.8 were malignant but no SUVmax cut-off was clearly identifiable to distinguish malignant from benign lesion on the ^18^FDG-PET/CT.

Once the ^18^FDG-PET/CT was done, further investigations in our hospital (CHUS) were not performed in 89 of the 304 patients (29.4%) with thyroid incidentalomas. Reasons varied from refusal of additional workup, lost at clinical follow-up to extensive disease with a critical vital prognosis. In fact, more patients were deceased in the «No additionnal work up» group [51/84 (60.7%)] compared to the group «With additional work up» [62/200 (31.0%)] (*p* < 0.001). We also noted that patients without additional investigations had thyroid incidentaloma’s SUVmax and size values inferior to those who undergo additional workup (*p* < 0.001 and *p* = 0.002 respectively) (Table 2).

**Table 2:** Follow-up of patients with thyroid incidentalomas detected on 18FDG-PET/CT at the CHUS

Characteristics	No additional work up (*n* = 84)^a^	With additional work up (*n* = 200)^a^	*p*
Deceased/Alive^a^	51/33	62/138	<0.001
SUVmax	1.5 [0.0–3.0]	3.3 [2.1–4.6]	<0.001
Size (cm)	1.0 [0.8–1.4]	1.3 [1.0–1.7]	0.002

^a^Data on deceased/alive status were available for 84/89 patients without follow-up and 200/215 patients with follow-up

Data are shown as Mean ± standard deviation, Median [IQR] unless otherwise indicated

## Discussion

Thyroid incidentalomas and PET/CT inspired a large amount of clinical research recently as this nuclear medicine tool for imaging increased in popularity for cancer diagnosis and follow up. Previous studies reported a detection rate for thyroid incidentalomas identified with ^18^FDG-PET/CT of 0.1 to 4.3% [5–12, 15], and a risk of malignancy between 10.3 to 80.0% [5–12] (Table 3). At our institution, the prevalence of incidentalomas was 0.74%, which is similar to the data of studies that included a large number of patients, like ours [5, 6, 11,  16]. A recent systematic review and meta-analysis published in 2014 by Nayan et al. stated that the pooled proportion of malignancy was calculated as 19.8%. Thirty-one studies were included in this review for a total of 197,296 PET studies and 3659 focal thyroid incidentalomas [4]. In our cohort, more than half of the operated patients (25/46) were diagnosed with malignancy. This represents a rate for malignancy of 54.3% in patients who underwent a lobectomy or a thyroidectomy. This high rate should not be interpreted as a rate of malignancy for TI knowing that a majority of patients with TI (*n* = 258) do not have a final histopathological result. Some studies [5–12, 15] have reported malignancy risk based on the surgical patients alone, which is a misinterpretation since these specific studies had a minority of patients brought to surgery for definitive diagnosis. From our data, we can then propose that the minimal rate of malignancy for thyroid incidentalomas is 8.2% (25/304) in this specific cohort. Even if we extrapolate and include the non-surgical patients with FNA results of Bethesda 6 (malignant), this rate rises to 9.2%, which is clinically unsignificant. In contrast, if we use the 61 nonsurgical patients with FNA results of Bethesda 2 (benign) and the 21 surgical patients with a “Benign” final histopathological result, we can again extrapolate that 26.9% of the TI were benign. Thus, the rate of malignancy has to lie between 8.2 and 73.1%. Nevertheless, this value (8.2%) is certainly compatible with an underestimation of the real malignancy rate in TI and the exact value stays unrevealed in this cohort.

**Table 3:** Characteristics of previous studies on thyroid incidentalomas identified with 18FDG-PET/CT or PET alone

Study	Year	Study Type	Technique	Patients	Incidentalomas *n* (%)	Histopathologic confirmation	Risk of malignancy % (*n*)
Jamsek et al. [15]	2015	Retrospective	PET/CT	5911	230 (3.89)	18	15.2 (10/66)
Pagano et al. [6]	2011	Retrospective	PET/CT	10 881	191 (1.8)	52	28.9 (15/52)
Yang et al. [5]	2012	Retrospective	PET/CT	15 948	395 (2.5)	43	29.5 (43/146)
Kao et al. [10]	2012	Retrospective	PET/CT	942	21 (2.2)	6	50 (3/6)
Nilsson et al. [8]	2011	Retrospective	PET/CT	3641	64 (1.8)	27	25 (16/64)
Ohba et al. [9]	2010	Prospective	PET	1501	20 (1.3)	20	55 (11/20)
Kim et al. [16]	2010	Retrospective	PET/CT	11 623	159 (1.4)	159	23.3 (37/159)
King et al. [11]	2007	Retrospective	PET et PET/CT	15 711	22 (0.1)	22	13.6 (2/22)
Yi et al. [12]	2005	Retrospective	PET/CT	140	6 (4.3)	5	80.0 (4/5)

Our second objective was to determine a cut-off value for the SUVmax. In general, malignant lesions have higher glucose metabolism than benign lesions, hence a higher SUVmax value. Similarly to Yang et al., we were not able to determine a specific SUVmax cut-off value that could offer a distinction between benign and malignant lesions [5]. In fact, in our study, malignant lesions had higher mean SUVmax values than benign lesions, but the difference was not statistically significant (*p* = 0.499). In addition, one patient was probably operated on the sole basis of a high SUVmax value (SUVmax = 55), since the cytology and echography results were intermediate and low risk respectively. Histopathological report confirmed the presence of a Hurtle cell follicular carcinoma. This is consistent with the proposition where lesions with frankly elevated SUV values present an increased risk of malignancy, but a cut-off value has yet to be determined. A recent Meta-analysis reported a cut-off value for the SUVmax of 3.3 [17]. Indeed, the authors state that this value has a good sensitivity (82.4%) but could bring a high proportion of false negative (specificity = 36.8%), probably related with an undeniable overlap in the SUVmax between benign and malignant thyroid incidentalomas. In the same study, half of the study pooled did not have a difference statistically significant of the mean SUVmax for malignant vs benign lesions. This confirms how inconsistent are the values in the published series and warrant further studies.

Literature shows a great amount of variability in the management of patients with thyroid incidentalomas, making it a dilemma for clinicians. More recently, guidelines from the American Thyroid Association recommended that all sonographically confirmed thyroid nodule >1 cm incidentally discovered on ^18^FDG-PET/CT should be biopsied with an FNA [13]. Moreover, this study tried to shed some light on TI management, especially for patients medically fit for surgery, but some degree of uncertainty still persists since nearly a third (29.4%) of our patients did not get any further evaluation. Table 3 shows that the insecurity of their prognosis influenced the decision in regards of TI investigation while more patients were deceased in this group than in the group with further investigations.

Despite the lack of a prospective cohort, we tried to propose a practical management algorithm for thyroid incidentalomas discovered with ^18^FDG-PET/CT. According to our data, it was difficult to simplify the ATA recommendation on this specific clinical problem. The cytoponction, in our study, had a strong correlation with final histopathological result in surgical patients. It certainly has to remain the key element in the management of those lesions. Emphasis should be put on a comprehensive thyroid physical examination and an evaluation of the vital prognosis of the patient related to the underlying disease. This will help the clinician to decide if it is still pertinent to refer the patient for further investigations, at least a cytoponction and a neck ultrasound.

### Limitations of the study

Some limitations are worth mentioning. First, the retrospective nature of our study. Second, the selection bias that underestimates the prevalence of thyroid incidentalomas. In fact, many patients had a fortuitous lesion identified on the ^18^FDG-PET/CT that was not possible to confirm with their chart because we selected the population of patient treated in our center in Sherbrooke. A centralised electronic record available throughout the province would have overstep this bias. Third, like many other studies done previously, we do not have histopathologic confirmation for the vast majority of TI for reasons mentioned above.

## Conclusion

Thyroid incidentalomas detected with ^18^FDG-PET/CT are relatively infrequent, but the potential risk of malignancy remains elevated. Fine needle aspiration biopsy is the investigation of choice to rule out a malignant incidentaloma when there is no other element in the clinical portrait to preclude such additional work up. Clinicians should keep a high index of suspicion for TI, while more than half of patients who underwent surgery received a malignant diagnosis. More prospective studies are needed to confirm a valid SUVmax cut-off value and to add some useful information for TI management.

## Abbreviations

^18^FDG-PET/CT: ^18^F-fluorodeoxyglugose Positron Emission Tomography with computed tomography
ATA: American Thyroid Association
CHUS: Centre Hospitalier Universitaire de Sherbrooke
FNA: Fine-needle aspiration
SUVmax: Maximum Standardized uptake value
TI: Thyroid incidentaloma
